# Closure of the Peritoneum in Laparoscopic Transabdominal Preperitoneal Inguinal Hernia Repair (TAPP) With Cyanoacrylate Glue in a Microdroplet Device: A Single Surgeon Prospective Comparison vs. Barbed Suture

**DOI:** 10.3389/jaws.2024.12562

**Published:** 2024-05-01

**Authors:** Juan Manuel Suárez-Grau, Laura Navarro-Morales, Luis Tallon-Aguilar, Salvador Morales-Conde, Francisco Javier Padillo-Ruiz

**Affiliations:** ^1^ Virgen del Rocío University Hospital, Seville, Spain; ^2^ Quirónsalud Sacred Heart Hospital, Seville, Spain; ^3^ Virgen Macarena University Hospital, Seville, Spain

**Keywords:** closure peritoneum, TAPP, hernia, laparoscopy, glue

## Abstract

**Purpose:**

To describe and compare a peritoneal closure technique using cyanoacrylate glue (Glubran 2^®^, GEM, Cardiolink SL) with a microdroplet device (Glutack^®^, GEM, Cardiolink SL) in laparoscopic transabdominal preperitoneal repair (TAPP) of inguinal hernia with the routinely used barbed suture peritoneal closure (V-Lock 3.0, Covidien France).

**Materials and methods:**

From January to August 2022, 60 patients undergoing TAPP repair for uni- or bilateral inguinal hernia were randomized into one of two groups. One using as mesh fixation and peritoneal closure the Glutack^®^ device with Glubran 2^®^ cyanoacrylate glue (Glu-close group) and the other using mesh fixation with cyanoacrylate and peritoneal closure with V-lock 3.0 (Sut-close group), with a follow-up of 12 months. Demographic variables, operative time, peritoneal closure time, main surgical findings and main intra- and postoperative complications were analyzed prospectively.

**Results:**

63 patients were included with no losses to follow-up. The mean operative time was 34 min (range 58.25) for the glu-close group and 40 (range 64.25) for the sut-close group, with no conversion (0%) for either group. The mean flap closure time was 1.18 min (SD 24 0.19) for the glu-close group and 3.24 min (SD 0.78) for the sut-close group, with statistically significant differences (*p* < 0.001). The intraoperative complication rate was 0 for the glu-close group and 0 for the sut-close group, with no significant difference. The median hospital stay was 0.8 days (range, 0–1) for both groups. The median duration of follow-up was 12 months and none had hernia recurrence. The postoperative VAS score at the first and second check-up at 1 month and 3 months was 2.83 (SD 1.341) and 0.60 (SD 0.621) in the sut-close group and 1.03 (0.984) and 0.24 (SD 0.435) in the glue-close group, with significant differences (*p* < 0.001 and *p* < 0.012).

**Conclusion:**

The data demonstrated by the study are that the glue can be used safely to close the peritoneum and that the method provides a small, statistically significant but not clinically relevant reduction in the time to close the peritoneal flap, as well as in postoperative pain after surgery in short and medium term.

## Introduction

Inguinal hernia repair is one of the most common general surgical procedures performed worldwide. Although laparoscopic inguinal hernia repair was initially indicated for bilateral inguinal hernias or recurrences in open anterior hernia repair procedures, it has become the first choice for most patients, reaching a very important globalization. The potential benefits of the laparoscopic approach with early postoperative recovery and a possible decrease in the incidence of long-term groin pain are well known [1].

The introduction and improvement of new materials has contributed greatly to their development, facilitating their spread among professionals and medical centers. One of the materials in which much progress has been made is the fixation of meshes with glue, changing the old paradigm from the recurrent use of traumatic fixation to the use of atraumatic fixation with glues [2].

The closure of the peritoneal flap during the TAPP technique has also been and remains a point of controversy among professionals.

Selecting the most appropriate technique for inguinal hernia repair is a challenge. The best surgical technique should achieve a low risk of complications (pain and recurrence), (relative) ease of learning as well as reproducibility among professionals, and rapid recovery.

This is the first randomized control trial of the use of the glubran cyanocrylate-device glue device for peritoneal closure in laparoscopic TAPP-type groin hernia repair. The aim of this study is to evaluate the use of the Glubran 2^®^ cyanoacrylate-derived glue with the Glutack^®^ microdroplet device for peritoneal closure in laparoscopic TAPP-type inguinal hernia surgery in comparison with the standard closure with barbed suture.

## Material and Methods

This is a multi-center comparative prospective single surgeon study consisting of laparoscopy inguinal hernia treatment patients requiring inguinal hernia surgery using the TAPP technique. Patients were included if they met the following requirements:

Unilateral or bilateral inguinal hernia with one or more of the following characteristics: 1) previous infraumbilical surgery, 2) incarcerated hernia, 3) obesity, 4) female patient, 5) recurrent inguinal hernia with mesh. Eligible patients were approached, and informed consent was taken.

All other patients were selected for the TEP technique or, in case of contraindication of general anesthesia, they were indicated for open inguinal hernia surgery, therefore they were not included in the study. Large L3 or M3 hernias were not included in the study, as they require traumatic fixation (the exclusion criteria).

The study was completed by including 63 patients with L1, L2, M1, M2 uni or bilateral inguinal hernia undergoing TAPP (the European hernia society groin hernia classification).

Thirty patients were randomized (by a simple random sampling method) to the Glu-close group (case group): peritoneal closure with the Glutack device^®^ and 33 patients were randomized to the Sut-close group (control group): after mesh placement, peritoneal closure was performed with absorbable 3.0 barbed suture. The main intraoperative findings and postoperative results were analyzed prospectively. Patients were analyzed with a minimum follow-up of 12 months.

The surgeries were performed by a single senior surgeon with considerable experience in laparoscopic abdominal wall repair. Patients were included in the control group with Vlock 3.0 absorbable barbed suture closure (Sut-close group) or case group with cyanoacrylate-derived microdroplet device (Glu-close group) respectively according to a simple randomisation method. All patients were transferred to the awakening room for immediate recovery and were discharged on a short stay basis (less than 24 h).

All patients were given postoperative recommendations with prescribed conventional analgesia, relative rest for 1 week and follow-up in 1 and 3 months in outpatient consultations with the same surgeon who performed the surgery.

The following variables were taken into account: demographic variables (age, sex, BMI, ASA, type of hernia according to the EHS) [2, 3], intraoperative variables (operative time, surgical conversion, intraoperative complications, flap closure time), postoperative variables hospital stay, postoperative pain according to the pain rating visual analogue scale (VAS) at 1 and 3 months, as well as seroma, haematoma, bleeding and recurrence rates. Randomization was performed using a computer-generated program. SSPS 29 was used was used for statistical analysis. As the samples were very homogeneous, the design of the study was similar to that of the literature, using groups of patients to perform analyzes with a 95% confidence interval and a 5% margin of error. Because it would be easy to find important differences in the groups, a sample n of approximately 30 patients per group was calculated. Levene’s test was used to ensure the similarity of variances. The Student’s T was used for the quantitative variable analysis to compare both groups (peritoneum closure time and VAS for pain). In the analysis of the qualitative variable (bleeding, peritoneum rupture…) the Fischer’s exact test was performed.

**FIGURE 1:**
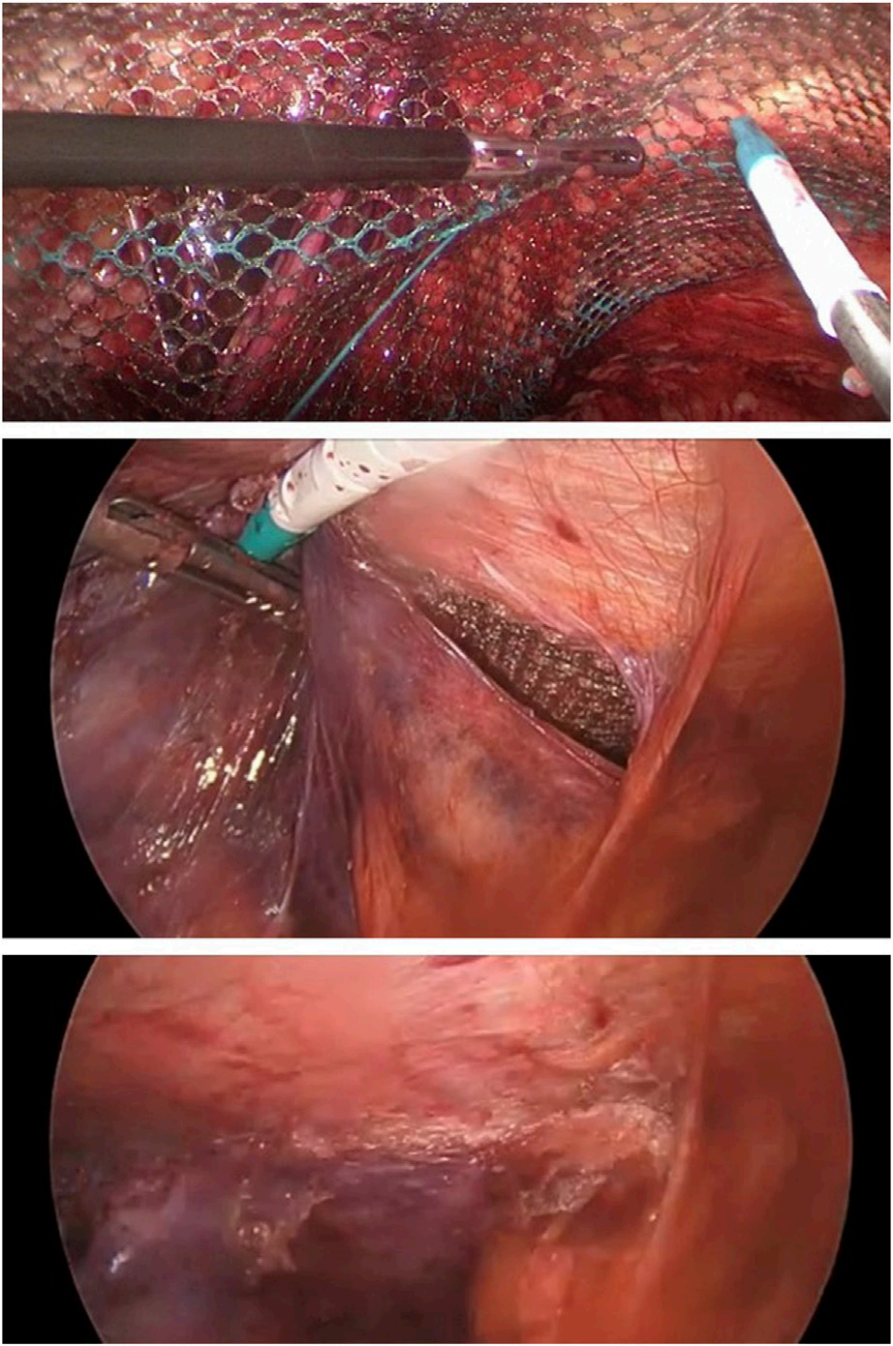
Atraumatic fixation in Cooper's Ligament, Closure of Peritoneum an final view of the peritoneaum closed by glue.

Procedure: Surgery is performed under general anesthesia with pneumoperitoneum established through a Veress needle at 15 mmHg in the left hypochondrium. Subsequently, an 11 mm optical trocar is introduced at the umbilical level and two 5 mm trocars at the level of the crossing of the bilateral supraclavicular midline with the umbilical level. The peritoneum is opened 4 cm above the inguinal orifice and the Retzius and Bogross spaces are dissected. Subsequently, the hernial content is reduced, with lateral hernias (L1 or L2) being indirect and medial hernias (M1 or M2) being direct. The vas deferens is then medialised, separating it from the spermatic vessels (in the case of males), or the round ligament is dissected (in the case of females). A three-dimensional PDVF polymer mesh (Dynamesh Endolap 3D, Cardiolink S.L.) is placed and fixed using Glubran 2^®^ (N-butyl-2-cyanoacrylate + Methacrylosisolfolane co-monomer), which polymerises rapidly and has a low-temperature exothermic reaction, in Cooper’s ligament and at the lateral end of the mesh Figure 1.

After mesh placement, the pneumoperitoneum pressure is lowered to 10 mmHg and the peritoneum is closed.

## Compliance With Ethical Standards

Disclosure of potential conflicts of interest: none of the authors have any conflict of interest to declare.

Research involving Human Participants: the Direction committee of both centers have approved the original research study.

Informed consent: all patients signed the document for inclusion in this study. All patients have been informed about the surgery and the potential complications and results of the intervention.

## Results

From January to August 2022, a total of 130 patients with groin hernia were recruited to the study. After monitorization, 67 patients were excluded from the analysis for not meeting the requirements of the study protocol. 63 patients with laparoscopic transabdominal-preperitoneal (TAPP) inguinal hernia repair were included in the study, the baseline clinical characteristics of which are summarized in Table 1.

**TABLE 1:** Patients characteristics.

	TAPP (n-63)	
	Glu-cose (n = 30)	Sut-close (n = 33)
Age (y), median (IR)	45	42
BMI (kg/m^2^), median (IR)	31	29
Sex (male/female)	17/13	16/17
ASA (n)		
I-II	24 (80%)	27 (81%)
III	6 (20%)	6 (19%)
EHS (n)		
L2	18 (60%)	23 (68.5%)
M2	12 (40%)	10 (30.5%)

ASA, indicates American Society of Anesthesiologists, BMI: body mass index, IR: interquartile range, EHS: european hernia society, TAPP: trans abdominal preperitoneal.

There were 16 males and 17 females for the control group and 17, 13 for the experimental group, with a mean age of 42 (SD 40) for the control group and 45 (SD 30.43) for the experimental group. The mean BMI was 29 (SD 27.32) for the control group, and 31 (SD 30.2) for the experimental group. The ASA anesthetic risk (I, II and III) for the control group was I (20%), II (70%), III (10%) respectively, while for the experimental group it was I (10%), II (70%) and III (20%). The type of hernia according to the EHS classification was L2 (82%), M2 (20%) for the control group, and L2 (60%), M2 (40%) for the experimental group. The groups were homogenous using Levene’s test for the variances.

In terms of mean operative time, surgery lasted 40 min (SD 64,25) with no conversion in any case (0%) for the control group and 34 min (SD 58,25) with no conversions for the experimental group. The mean peritoneal flap closure time was 3.24 min (SD 0.78) for the sut-close or control group and 1.18 min (SD 0.19) for the glu-close or experimental group, with statistically significant differences Supplementary Figure S1.

The intraoperative complication rate was 0 for the glu-close group and 0 for the sut-close group. There was only one minor bleeding (controlled with cautery) in the sut-close group. There was only one peritoneal tear with barbed suture, which required the use of a second suture to complete the sealing of the peritoneum, with no significant differences. The median hospital stay was 1 day (range, 0–1). The median duration of follow-up was 3 months (range, 3–5) and there were no recurrences (0%) for either group at one or 3 months. The postoperative VAS score at the first and second follow-up at 1 and 3 months was 2.83 (SD 1.341) and 0.60 (SD 0.621) in the sut-close group and 1.03 (0.984) and 0.24 (SD 0.435) in the glue-close group, with statistically significant differences *p* < 0.001 (at 1 month) and *p* < 0.012 (at 3 months). There was no loss to follow-up at 12 month. The postoperative VAS score at the 12 months was 0 in the glu-close group and 0 in the sut-close group, in the same way as in the percentage of recurrence (Table 2).

**TABLE 2:** Intrapeoperative and postoperative outcomes.

	TAPP (n-63)		
	Glu-cose (n = 30)	Sut-close (n = 33)	p-valor
Mean operative time min (SD)	34	40	NO differences
Conversion to open surgery, n (%)	No	No	NO differences
Mean close of peritoneum time (SD)	70.79 (11.65)	194.30 (46.68)	***p* < 0.001**
Mean hospital length of stay days (SD)	0.8	0.8	NO differences
Intraoperative complications			
Controlled Bleeding in the line of peritoneum	No	2	***p* = 0.046**
Rupture of peritoneum	1	2	*p* = 0.601
Overall postoperative complications, n (%)			NO differences
Bleeding	No	No	
Wound hematoma	No	No	
Wound seroma	10%	9%	
Recurrence, n (%)	0 (0%)	0 (0%)	
Chronic pain, mean VAS n (%)			
1st month	1.03 (0.984)	2.83 (1,341)	***p* < 0.001**
3rd month	0.24 (0.435)	0.60 (0.621)	***p* < 0.012**

TAPP: trans abdominal preperitoneal.

Bold values represent the statistical significance.

## Discussion

In our study we can observe that the use of glue is safe to close the peritoneum without higher percentages of recurrence in the group with glue vs. the use of suture. Likewise, at a clinical level, there are no differences in terms of operative time or postoperative pain.

Laparoscopic inguinal hernia repair is rapidly becoming an alternative procedure to the standard open approach and its advantages are well established. Knowledge of the anatomy of the surgical field and the further development of new tools make this surgery simpler, faster and safer. However, the type of mesh used, its fixation and the peritoneal closure for the TAPP technique remain the issues still under discussion [1–3].

If we focus on improving the results obtained from the laparoscopic inguinal hernia era, we are talking about trying to reduce acute and chronic postoperative pain without increasing hernia recurrence. The two main topics of development in this respect are the emergence of new meshes and new fixation systems. For this reason we propose this study in which the use of an alternative closure with glue versus the traditional use with suture is studied. During the last decade, the development of the procedure for laparoscopic inguinal hernia repair has changed considerably. There are several methods of mesh fixation, such as: tackers, staples, self-fixation, fibrin sealants, glues and sutures. However, there is no consensus on which method is best; although atraumatic methods are known to be safe and effective, their use depends on the surgeon’s preference.

The detection of persistent postoperative pain after traumatic mesh fixation, together with the discovery of the “triangle of death” and the “triangle of pain,” has led to the recommendation of not using traumatic fixation as standard (B grade recommendation), except in specific case such as hernia recurrences and M3 and L3 type hernias, and in case of need, to avoid its use on the lateral aspect of the mesh, avoiding the risk of lateral femoral cutaneous nerve entrapment [2].

Some authors, aiming to reduce postoperative pain, report their experience avoiding the use of traumatic fixation and securing the mesh only with glue, such as fibrin glue or cyanoacrylate. In a meta-analysis published in 2016 with 1,454 patients undergoing laparoscopic hernioplasty, it was found that chronic pain was significantly less frequent in those patients treated with fibrin glues or *cyanoacrylates* than using traumatic methods [4]. However, controversy still exists regarding the optimal way to close the peritoneum after using the TAPP technique [5].

The more rapid method of peritoneal closure using helical staples is contraindicated, assuming a higher risk of nerve injury and bleeding. Currently, the most commonly used alternative is continuous suturing. However, suturing the peritoneum is not as simple as it seems and remains a challenging maneuver that requires specific surgical skills to avoid tears or rupture that may expose the mesh to the bowel, with secondary obstruction or fistulation. The development of barbed sutures has decreased the difficulty of this procedure; however, peritoneal ruptures still occur, especially in cases where the hernia sac reduction maneuver has been particularly difficult (e.g., with sliding hernias, where the peritoneum of the flap is too thin and too weak to be closed with a continuous suture). For these reasons, we believe that the key advantage of the technique described here is particularly for those cases where the peritoneum is at increased risk of tearing or rupturing during closure [6, 7].

In addition, continuous suturing of the peritoneum does not avoid the risk of nerve entrapment, as the suturing of the upper peritoneal flap often includes part of the abdominal wall. Therefore, in our view, the simplest and least expensive technique to close the peritoneum may be glue, which has been investigated in few studies to date.

The most frequently studied adhesive product is N-2-butyl cyanoacrylate, which shows excellent capacity for both peritoneal and mesh closure, which is achieved after only a few seconds. However, being a non-biological glue, one of the main criticisms is that when this product is in contact with the intestine, adhesions can develop. However, the study by Wilson and Hickey [5] which recently investigated their experience with polypropylene mesh and peritoneal closure reported excellent results with no long-term complications, but considerable precautions are required when using this product to prevent debris from falling out and coming into contact with the bowel. An alternative to cyanoacrylate is the biological fibrin homologue glue (*Tisseel, Baxter Healthcare*) which has both a sealant and haemostatic function. In contrast to cyanoacrylate, the adhesive strength is not as optimal, having lower tensile strength, making cyanoacrylate a safer tool for closing the peritoneum [4, 7–9].

To our knowledge, only one study has investigated its use in both mesh fixation and peritoneal closure for the TAPP technique, with favorable results. After the final of the study we have closed the peritoneum with the method described in more than one hundred patients with the same results of the study.

The results of our study show the safety of the procedure and it should be considered as a reliable and easy to perform method in the TAPP procedure. Since it is easy to use as an alternative to conventional suturing, it seems subjectively faster to perform although without statistically significant differences. But it is also safe without causing higher recurrence rates or pain in the short and medium term.

## Study Limitations

Short follow-up: bearing in mind that the follow-up is 3 months postoperatively, we assume that the main variables to be studied are based on postoperative complications within this time range, without being able to provide data on recurrences after this time.

The experience of a single surgeon may also carry a bias limiting the ability to generalize this experience to a group of professionals.

## Conclusion

Material development is crucial for further advancement of laparoscopic inguinal hernia surgery. Adhesives are making it possible to perform faster and more precise fixation procedures compared to older sutures. The data demonstrated by the study are that the glue can be used safely to close the peritoneum and that the method provides a small, statistically significant but not clinically relevant reduction in the time to close the peritoneal flap, as well as in postoperative pain after surgery. Short and medium term.

## Data Availability

The raw data supporting the conclusion of this article will be made available by the authors, without undue reservation.

## Supplementary material

SUPPLEMENTARY FIGURE S1 Time comparison in closing time of the peritoneum.

SUPPLEMENTARY VIDEO S1 Closure of the peritoneum in laparoscopic TAPP with cyanoacrylate glue in a microdropet device: a single surgeon prospective comparison vs barbed suture.
