# Supplementary figures and images for: Closure of the Peritoneum in Laparoscopic Transabdominal Preperitoneal Inguinal Hernia Repair (TAPP) With Cyanoacrylate Glue in a Microdroplet Device: A Single Surgeon Prospective Comparison vs. Barbed Suture
Source: J Abdom Wall Surg. 2024 May 1;3:12562. doi: 10.3389/jaws.2024.12562 (PMC11100424; doi:10.3389/jaws.2024.12562)

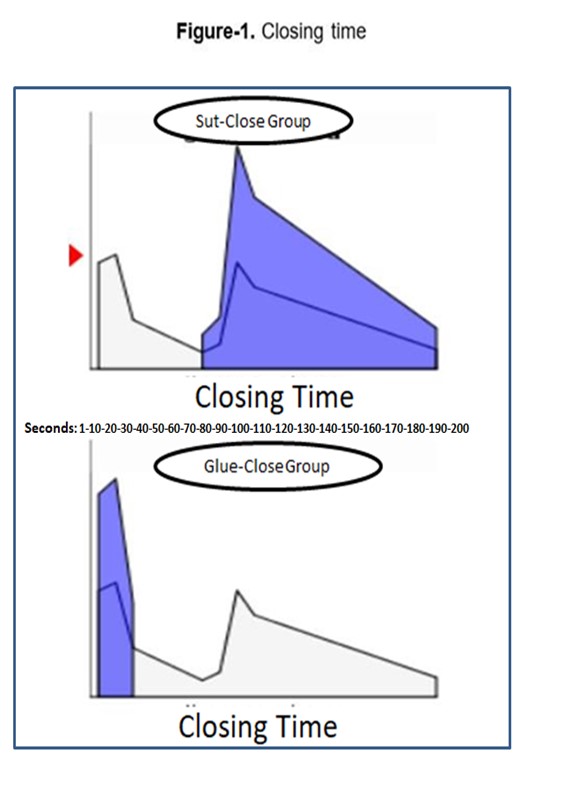

Supplement: Supplementary file 1 [file Image1.JPEG]
